# Latest Concepts in Endodontic Management of Pregnant Patients

**DOI:** 10.1155/2023/9714515

**Published:** 2023-09-26

**Authors:** Maryam Kuzekanani

**Affiliations:** Department of Endodontics, Kerman Dental School, Kerman University of Medical Sciences, Kerman, Iran

## Abstract

Pregnant patients and how to manage their treatments is one of the most important challenges in endodontic practice. Endodontic treatment on pregnant women is sometimes an emergency condition to control toothache due to irreversible pulpitis and odontogenic infection. Tooth decay, oral health, local and general anesthesia, analgesics, antibiotic prescription, drug interactions, and X-ray radiation are the most important considerations that may impact treatment planning and endodontic practice in pregnancy. The aim of this article is to notify and explain the latest concepts in the endodontic management of pregnant patients.

## 1. Introduction

Normally, during pregnancy, several changes occur due to the secretion of pregnancy hormones and their release into the bloodstream [1]. Polypeptides and steroids secreted from the placenta, also the pituitary, thyroid, and adrenal glands cause changes in the nervous, muscular–skeletal, cardiovascular, genital, and oral cavity systems functions and expose the pregnant mother to an increased risk of some infections and inflammations [2]. Among the changes related to oral and dental tissues during pregnancy, the following alterations can be mentioned.

### 1.1. Background Changes

Increase in the volume of plasma and blood cells, increase in heart rate [3], decrease in digestive tract movements [4], increase in glomerular filtration, increase in substance clearance [5], metabolic syndrome-like fuel changes [6], immunity response suppression [7], increasing estrogen, and progesterone receptors and subsequently increasing cell division and cell differentiation [8], increasing the activity of osteoclasts are important background changes seen in the pregnancy [9].

### 1.2. Physiological Events

Pregnancy gingivitis [8], pyogenic granuloma [10], recurring aphthous lesions [11], change in the microbial load of the oral cavity [12], change in the flow and secretion of saliva [13], and increased tooth movement [14] are the physiologic events during pregnancy.

### 1.3. Pathological Events

Potential dermatoses and tumors affected by pregnancy, pregnancy-specific dermatoses [15], tooth decay [16], tooth surface loss [17], and periodontal diseases [18] are the pathologic events that may threaten the health of the developing fetus and mother in pregnancy and reduce the quality of life of the mother as well as the fetus significantly [19].

Overall, studies have shown that oral and dental changes during pregnancy could be related to complications, such as pregnancy poisoning and other metabolic and mental changes during this period [20].

Considering the extensive changes in the mother's body during pregnancy and the impact of these changes on the health and quality of life of the mother and the fetus, dealing with pregnant women is one of the most important challenges in endodontic practice [21, 22].

The reason stated above is that pregnancy is accompanied by many changes, and along with these changes, environmental factors such as consumption of sugary substances, vomiting and as a result increased oral acidity, poor oral and dental hygiene due to impatience, and the risk of nausea and vomiting after brushing the teeth and using mouthwash, can increase the risk of tooth decay and pulp involvement in pregnant mother [23–25].

Despite the past, nowadays, local anesthetic injection in pregnancy is considered harmless because of the lack of evidence on adverse teratogenic effects in this period. However, it is better to use anesthetic solutions that have been categorized under Group B rather than those categorized in Group C to provide more conservative endodontic treatments during this condition [26, 27]. According to the The Food and Drug Administration (FDA) table of anesthetic solutions used in pregnancy, the anesthetic solutions of choice during this period are lidocaine and prilocaine, which are both categorized in Group B [27]. Pregnant women may choose to postpone taking radiographs until the end of the first trimester since this time frame is the most crucial for the fetus's development. There is not any medical evidence for taking this decision, but it may provide peace of mind.

Since the ethical issues inhibit doing direct studies on the fetus, most of the history and beliefs regarding the dental X-ray pathologic impacts on the fetus originate from observations in patients who were accidentally irradiated by Japan's Hiroshima nuclear explosion as well as the Chernobyl nuclear disaster. According to the observations of the victims of these high levels of radiation exposure, the consequences of the exposure to radiation can be categorized into four groups: pregnancy loss, malformation, retardation, and carcinoma [28]. Taking dental X-rays may be postponed until after the delivery, but this plan is also not recommended where taking X-rays is critical to diagnose dental issues that could become challenging if they are not detected and treated [27–29].

In order to manage pain, prescribing acetaminophen is preferable to ibuprofen and Novofen because the last two may increase the risk of bleeding and late delivery [29]. Administration of corticosteroids and tetracycline, NSAID, morphine, and aspirin are prohibited in pregnant women, and the antibiotics of choice for pregnant women are amoxicillin and clindamycin [30]. In case of any necessary X-ray irradiation, using an apron and a lead necklace is suggested to provide peace of mind. Although the treatment of pregnant mothers is associated with some complications and difficulties, however, dental treatment is an important and inevitable part of the care plan for pregnant women, and the management of these patients requires proper timing, duration of treatment management, use of anesthetics, and prescription of appropriate drugs [31].

Pregnant women may use over-the-counter analgesic drugs and antibiotics to control pain and pulp infection, and using these drugs instead of dental treatment is associated with harming the health of the mother and fetus [28].

Due to the possible lack of awareness about pregnancy complications and systemic interactions, and in order to avoid responsibility for unpleasant and unwanted events, the endodontists are recommended to consult with the gynecologists regarding the necessity and time of treatment, anesthetic, analgesic, and antibiotic drugs type and the dosage of prescription before starting any root canal treatment.

However, in cases where the patient comes to the clinic with very severe and beyond the threshold of tolerance pain, it is impossible to comply with such an order. and it is necessary that the safest and most conservative emergency root canal treatment immediately starts after obtaining the medical and dental history, in order to control and treat the patient's pain. After controlling the emergency situation, the patient should be referred to the obstetrician and gynecologist to obtain permission and necessary consultations [32–34].

## 2. Oral and Dental Health in Pregnancy

Several studies have investigated the interactions between the mother's poor oral and dental health during pregnancy and pregnancy outcomes, and also the oral and dental health in the children of these mothers. These outcomes range from the risk of early tooth decay to premature delivery and low birth weight. In addition to personal compliance with oral and dental hygiene, pregnant mothers face other challenges to maintain optimal oral and dental hygiene [35], which include negative experiences and a lack of awareness of the value of dental health care [36].

## 3. Drug Considerations in Pregnancy

The biodistribution of the drugs changes widely during pregnancy. Reduction of maximum plasma capacity, reduction of plasma half-life, increase in solubility of lipids, and increase in drug clearance are among these changes [35–38]. This change in the dynamics of drugs in the bloodstream causes faster and easier release of drugs through the placenta that may end to teratogenic changes and birth weight loss, threatening the health of the fetus and also abortion. Due to the sensitivity of the fetus to the drugs used by the mother, it is recommended to refrain from taking the drug during the first 13 weeks of pregnancy and if necessary, the drug should be taken under the supervision of a specialist gynecologist considering the possible threats of the compounds.

## 4. Analgesics

Analgesic drugs are used in a limited dose and short timeframe to treat and reduce the pain of the pregnant mother. Acetaminophen is the most common and safest drug used during pregnancy, which is in Group B according to the classification of the American Food and Drug Administration. The most probable side effect of acetaminophen is liver toxicity, and due to the variety of formulations of this drug, pregnant women are advised not to consume more than 4 g of acetaminophen daily. Other analgesics such as Ibuprofen are placed in Group B during the second and third trimesters, but for the third trimester, they may be considered as Group D because it may cause a decrease in amniotic fluid volume, premature heart valves, closure, and failure to open the birth canal. The prescription of acetaminophen along with codeine or oxycodone is often without prohibition, but it should be noted that the long-term use of these drugs can affect the mental health of the developing fetus [39, 40].

## 5. Antibiotics in Pregnancy

Most of the common antibiotics in dentistry are in Group B of the American Food and Drug Administration (except gentamicin and doxycycline, which both belong to Group D). Reports show that gentamicin causes fatal ototoxicity and doxycycline and its derivatives cause teeth discoloration and weakening of growing bones. It has been shown that the broad-spectrum antibiotic; ciprofloxacin (fluoroquinolones) causes damage to the joints by leaving traces on the developing cartilage. Metronidazole is classified in Group B of the American Food and Drug Administration; its use is prohibited in the first trimester of pregnancy due to its teratogenic effects [41, 42].

## 6. General and Local Anesthetics in Pregnancy

The limited and conservative use of anesthetic drugs is without problems if the type and dosage of the drug are chosen correctly. Anesthetic drugs, such as lidocaine and prilocaine, are classified in Group B of the US Food and Drug Administration, but mepivacaine and bupivacaine are mostly classified in Group C. Epinephrine as the vasoconstrictor in anesthetic solutions can reduce uteroplacental blood pressure in case of intravenous injection, but if it is used in a safe dose, is not dangerous for the mother and the fetus. The characteristics of different local anesthetics that may be used in pregnancy are summarized in Table 1 [27].

The FDA has provided a classification system that classifies different drugs, including local anesthetic solutions, according to their possible harmful side effects. Drugs under Groups A and B are considered to cause no danger to humans. The difference between Groups A and B is whether the drug has been tested on humans or has not. Drugs whose teratogenic effects cannot be eliminated are considered under category C. Group D includes drugs with positive evidence of human fetal risk. Drugs in Group X are inhibited from being prescribed during pregnancy [27].

In general surgeries, the use of general anesthesia is without problems if the proper pressure of oxygen and carbon dioxide is correctly maintained, but the use of teratogenic factors that may cause premature birth must be prevented. Nitrous oxide is a safe and common anesthetic drug in surgical procedures, but due to its side effects, such as premature birth, abortion, and fetal defects, it is recommended to be used only in emergency cases during pregnancy; otherwise, its use should be postponed until the termination of pregnancy [39, 43–45].

## 7. Radiation during Pregnancy

During pregnancy, the radiation dose exposed to the mother and the fetus, though low, should be applied at the proper dose. Regarding taking radiography during pregnancy, the general rule for X-ray irradiations is similar to imaging for all patients in general; radiation exposure is as low as reasonably achievable [46]. According to Kelaranta et al. [47], the fetal dose levels without lead shielding were <1% of the annual dose limit of 1 mSv for any individual in the public. Although custom shielding limits the X-ray exposure-induced risk of breast cancer death for the pregnant mother and the exposure-induced risk of childhood cancer death for the unborn, the necessity for fetal and breast lead shielding was considered irrelevant. A more important conclusion derived from their findings is that pregnancy is never a reason to avoid or postpone an important diagnosed dental radiographic examination. Later, in 2021, Bahanan et al. [48] proposed that there is insufficient public awareness regarding the safety of dental imaging during pregnancy. Pregnant women have high unrealistically and false perceptions of the risk from dental imaging, and this misunderstanding may lead to anxiety and postponing of necessary dental treatment. Since the knowledge and awareness of pregnant patients have a direct effect on their attitude and performance toward dental care, it is mandatory to establish community awareness teams that aim to educate societies about radiation doses, safety, and necessary protective considerations. Endodontists must reassure pregnant patients about the safety of dental imaging in pregnancy and clarify its risks and benefits for them.

## 8. Conclusion

Upon clarifying the most important issues in pregnancy and explaining how to manage them, nonsurgical endodontic treatment is a safe and effective treatment plan in case of acute symptomatic pulpitis in pregnant women and should not be postponed to the cost of maintaining a painful tooth and consumption of multiple drugs during pregnancy. Dental radiographs can be taken at any trimester during the pregnancy while keeping the dose as low as reasonably achievable. Local anesthetic-solution injections, analgesic drugs, and antibiotics prescriptions have no limitations in any trimester of pregnancy in low doses based on the latest statements of the American Dental Association and the American Obstetricians and Gynecologists Societies. Consultant with the Gynecologists is highly recommended to better manage these patients if there is no time limitation due to the severity of the pain. Further investigations on the safety of all these important endodontic treatment issues during pregnancy are recommended to provide safer treatments for this important group of medically compromised patients.

## Figures and Tables

**Table 1:** FDA—maximum dosage of use of local anesthetic solutions.

Anesthetic solution	Maximum dosage (with vasoconstrictors) (mg/kg)	Maximum total dosage (with vasoconstrictors) (mg)	FDA category
Lidocaine	7	500	B
Prilocaine	6	400	B
Mepivacaine	7	550	C
Bupivacaine	—	90	C
Articaine	7	—	C
